# Thermal Sensation in Older People with and without Dementia Living in Residential Care: New Assessment Approaches to Thermal Comfort Using Infrared Thermography

**DOI:** 10.3390/ijerph17186932

**Published:** 2020-09-22

**Authors:** Charmaine Childs, Jennifer Elliott, Khaled Khatab, Susan Hampshaw, Sally Fowler-Davis, Jon R. Willmott, Ali Ali

**Affiliations:** 1 College of Health, Wellbeing and Life Sciences, Sheffield Hallam University, Sheffield S10 2BP, UK; jennifer@centralmedicalservices.co.uk (J.E.); k.khatab@shu.ac.uk (K.K.); s.fowler-davis@shu.ac.uk (S.F.-D.); 2 School of Health and Related Research (SCHARR), University of Sheffield, Sheffield S10 2TN, UK; s.hampshaw@sheffield.ac.uk; 3 Electronic and Electrical Engineering Department, University of Sheffield, Sheffield S10 2TN, UK; j.r.willmott@sheffield.ac.uk; 4 Sheffield Teaching Hospitals, National Institute for Health Research (NIHR), Biomedical Research Centre, Sheffield S10 2JF, UK; ali.ali@sheffield.ac.uk

**Keywords:** infrared thermography, cutaneous temperature, skin blood flow, dementia, body temperature, thermal sensation, thermal comfort, imaging, mapping, environmental temperature, frailty

## Abstract

The temperature of the indoor environment is important for health and wellbeing, especially at the extremes of age. The study aim was to understand the relationship between self-reported thermal sensation and extremity skin temperature in care home residents with and without dementia. The Abbreviated Mental Test (AMT) was used to discriminate residents to two categories, those with, and those without, dementia. After residents settled and further explanation of the study given (approximately 15 min), measurements included: tympanic membrane temperature, thermal sensation rating and infrared thermal mapping of non-dominant hand and forearm. Sixty-nine afebrile adults (60–101 years of age) were studied in groups of two to five, in mean ambient temperatures of 21.4–26.6 °C (median 23.6 °C). Significant differences were observed between groups; thermal sensation rating (*p* = 0.02), tympanic temperature (*p* = 0.01), fingertip skin temperature (*p* = 0.01) and temperature gradients; fingertip-wrist *p* = 0.001 and fingertip-distal forearm, *p* = 0.001. Residents with dementia were in significantly lower air temperatures (*p* = 0.001). Although equal numbers of residents per group rated the environment as ‘neutral’ (comfortable), resident ratings for ‘cool/cold’ were more frequent amongst those with dementia compared with no dementia. In parallel, extremity (hand) thermograms revealed visual temperature demarcation, variously across fingertip, wrist, and forearm commensurate with peripheral vasoconstriction. Infrared thermography provided a quantitative and qualitative method to measure and observe hand skin temperature across multiple regions of interest alongside thermal sensation self-report. As an imaging modality, infrared thermography has potential as an additional assessment technology with clinical utility to identify vulnerable residents who may be unable to communicate verbally, or reliably, their satisfaction with indoor environmental conditions.

## 1. Introduction

To experience a thermally comfortable indoor environment, an older person living in residential care relies almost entirely upon decisions made by others. This is typically the care home staff who will regulate the temperature of the communal spaces and bedrooms. For those residents with dementia, simple interventions to adjust the physical stimuli of light, noise and temperature can improve a person’s quality of life [1] experience [2] and behaviour [3]. Thermal comfort, therefore, becomes an important aspect of wellbeing and quality of life, which may require a different set of indoor thermal adjustments (including clothing) than required for active younger people.

A fundamental starting point is to understand the definition of thermal comfort; a condition of mind which expresses satisfaction with the immediate environment [4]. As a subjective experience, it may well differ amongst groups of people sharing the same environment at the same time.

The international standard, EN ISO 7730 [5] covering the evaluation of moderate thermal environments (developed in parallel with the revised American Society of Heating, Refrigerating and Air-Conditioning Engineers (ASHRAE, standard 55) specifies methods for measurement and evaluation of thermal environments. Thermal sensation is predominately related to heat balance and is influenced by physical activity, clothing and the indoor environmental factors of air temperature, mean radiant temperature, humidity and air velocity [5]. By seeking to provide a comfortable thermal environment for the majority, building and architectural sciences have led the way in finding solutions to achieve thermal comfort for the built environment. A subjective seven-point thermal sensation scale [4] developed originally from the work of Bedford [6] is completed by each individual. It is a scale widely used in thermal surveys and field trials [7] and forms the basis to calculate the average thermal sensation vote of large groups of individuals exposed to the same environment, along with an index of those dissatisfied i.e., people who vote feeling too hot, warm, cool or cold.

Whilst international standards are available, their use has largely been focused on determining thermal comfort of the workforce in offices and factories. Much less is known about thermal comfort in the old and very old living in residential care [8,9,10]. The need for new perspectives on thermal sensation and thermal comfort in older age is now appreciated [3,11,12,13] particularly given that the changes that occur in the nervous system associated with ageing leads to a decrease (‘blunting’) of thermal sensitivity and thermal perception [14] especially in response to cold stimuli [15] which is most pronounced at the extremities and follows a distal-proximal pattern [16]. Furthermore, due to a diminished cutaneous vasoconstrictor response to body cooling [17], older people may lose both their perception of the environment and their ability to conserve heat at the extremities. They may therefore (a) not perceive themselves as cold and (b) have a reduced physiological efficiency to conserve heat and are therefore at an increased risk of ‘symptomless cooling’ [14,18] putting the older person at greater risk of chilling or worse still, hypothermia [16].

As evidence emerges [11] that the existing analytical models for determination and interpretation of thermal comfort are not appropriate in older age, opportunities open up to investigate multidimensional approaches to thermal comfort, specifically of relevance to older people.

The aims of this study therefore, were to (i) identify the range of thermal sensation self-reports amongst groups of care-home residents, with and without dementia, sharing the same indoor environmental conditions (ii) use objective, long-wave infrared (LWIR) thermography to map extremity skin temperature and (iii) determine the correspondence between thermal sensation self-report and the extremity thermal map.

## 2. Materials and Methods

### 2.1. Study Design

Prospective observational feasibility investigation.

Inclusion Criteria: residents living in residential care aged 60 years or over.

Exclusion criteria: residents unable to communicate or lacking capacity to respond to simple questions.

### 2.2. Sample Size

As a feasibility study, the target population is 70 participants. Assuming attrition, the revised target is 60 participants. With this number, there will be 90% power, at significance level 0.05, to detect one value or unit (°C) change in temperature with 1.4 SD difference between the two groups.

### 2.3. Participants

Adults living in residential care homes within the South Yorkshire and Derbyshire counties of the UK were recruited over 12 months.

### 2.4. Screening and Recruitment Pathway

Older adults were invited to give their written informed consent to participate in the study after first reading the participant information sheet. Screening for capacity was undertaken by research nurses of the clinical research network (CRN) for the Yorkshire-Humber and Derbyshire National Health Service (NHS regions). The manager of each residence was first contacted and information was provided verbally and via leaflets for the care home staff to retain. If the manager was interested in the objectives of the study, a researcher returned to the care home to discuss the specific details of the study. The care home manager identified those residents considered to have capacity to give their own informed consent as a study participant using a ‘Noticeable Problems Checklist’. For those residents with a medical diagnosis of dementia, or those considered by the care staff to have fluctuating capacity, the relative (or independent clinician) was provided with study information, by letter, and asked to consider willingness to give their signed consent on behalf of the resident. Once consent had been obtained, a mutually convenient date for recruitment was made to obtain signed consent from the resident or appropriate authority.

### 2.5. Recruitment

On the day of study, and with consent obtained, a further screening for capacity was undertaken using the Abbreviated Mental Test (AMT) [19], a 10-point scale used as a guide to screening for dementia in those without a formal dementia diagnosis. A score below 8 indicates a level of cognitive impairment warranting ‘assignment’ to the dementia category (D). Residents with AMT score of ≥8 were assigned to a ‘no dementia’ category (ND).

### 2.6. Data Collection

#### 2.6.1. Demographic Data

Demographic data was collected to include, age, gender, ethnicity, years in residence, ‘handedness’.

#### 2.6.2. Frailty Assessment

The level of clinical frailty was assessed on a seven-point, clinical frailty scale (version 2007–2009 Dalhousie University, Halifax, NS, Canada) [20].

#### 2.6.3. Body Temperature Measurement

Body temperature was measured at the tympanum (T_tymp_) using a Thermoscan device (Model LF 40, Braun, Lausanne, Switzerland) before thermography commenced.

#### 2.6.4. Past Medical History (PMH) and Current Medications

For each older resident, a brief medical history was obtained along with co-morbid condition/s and medication history, including polypharmacy. Past medical history (PMH) and medication type may influence: (a) the individuals’ thermal ‘perception’ and (b) heat distribution at the extremities; the former influencing feelings of thermal comfort and the latter, the appearance of the heat signature (i.e., the appearance of the thermal map).

PMH with potential to affect thermal comfort perception includes thyroid dysfunction, impaired neurological perception (e.g., learning difficulties, stroke, dementia, including Alzheimer’s disease, peripheral neuropathy). A PMH likely to affect heat distribution at the extremities (and thus the thermal map) is linked to vascular compromise. Pre-existing conditions were documented and include all forms of diabetes, hypertension, vasculitis, peripheral vascular disease, heart failure and Raynaud’s disease. Current medication/s and dose was also documented with drugs having potential thermoregulatory effects assigned to the following categories: (a) vasoconstrictor effects, (b) vasodilator effects, (c) neurological effects, (d) diuretic effects.

#### 2.6.5. Indoor Environment

Residents were studied in groups of two or more sitting in their usual daytime communal room. Responses were sought under ‘real world’ conditions of the care home. The study was scheduled to commence at least one hour after breakfast. This gave sufficient time to complete imaging before lunch was served. Recommendations for measurement of ambient conditions (EN ISO 7730) [5] were made for air temperature, (T_a_ °C), relative humidity (RH%) and air velocity (m·s^−1^) measured using a Kestrel environmental monitor (Kestrel 3000, Kestrel Instruments, Boothwyn, PA, USA).

#### 2.6.6. Clothing

An approximation of clothing fabric insulation for each garment worn by the resident was made using available reference values [21]. Insulation of clothing is expressed as a ‘Clo’ unit. Clothing ‘ensembles’ were estimated from a weighted valuation:

0.676∑ Icl,i + 0.117(1)

where ∑Icl refers to the sum of individual clothing items worn. A pragmatic approach to clothing category produced three groups: 0–0.50 Clo, 0.51–1.00 Clo, >1.0 Clo for light, medium and heavy clothing ensembles respectively. A Clo score of 0 corresponds to a person, nude. A Clo score of 1.0, the value of clothing insulation needed to maintain a person in comfort, sitting at 21 °C with air movement, 0.1 m·s^−1^, relative humidity (RH) ≤ 50%, the example used being a person wearing a business suit [21].

#### 2.6.7. Imaging-Long Wave Infrared (LWIR) Thermography

**Figure 1:**
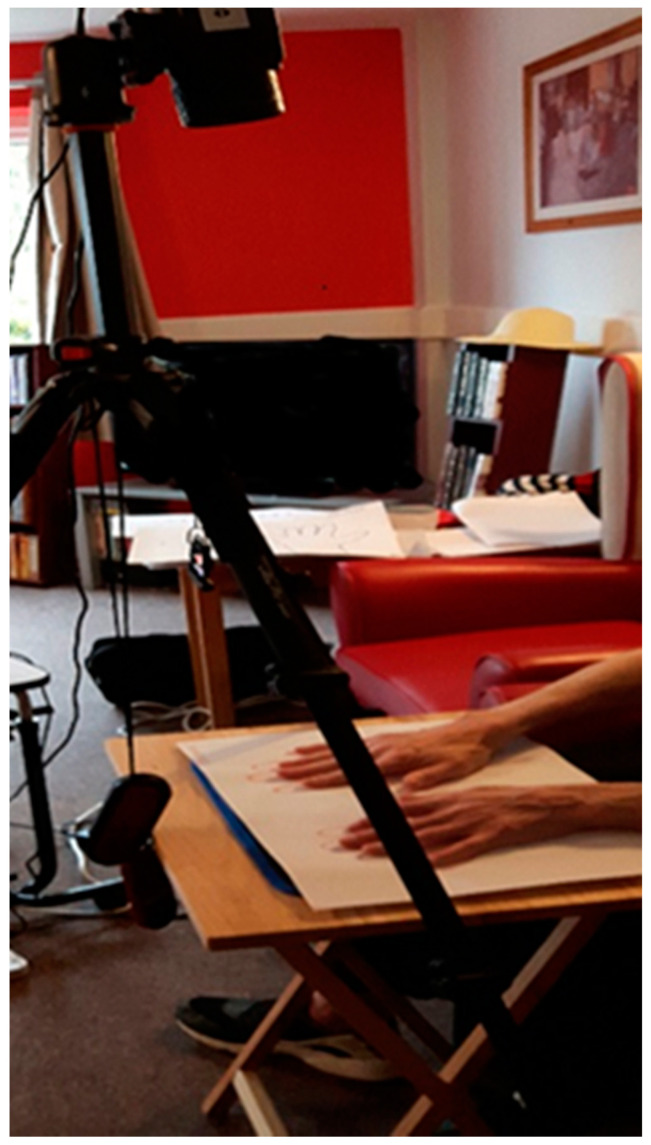
Long wave infrared imaging detector mounted on a tripod. The figure shows the participant during imaging with hands positioned upon a paper ‘hand template’ overlying an insulated tile. The portable table was used throughout the study to ensure consistency of the imaging set-up.

Infrared thermography was undertaken using an uncooled microbolometer detector, (model A-600 series, FLIR, Täby, Sweden) with image resolution 640 × 320 pixels, mounted on a tripod (Vanguard, Alta Pro 264AT, Dorset, UK) and connected to a laptop computer. The LWIR detector system was positioned such that the seated participants were comfortable. In the sitting position, acral region (both hands) were positioned, first with the dorsum followed by palmar surface upwards resting upon a paper ‘hand template’ (Figure 1) overlying an insulated tile.

**Figure 2:**
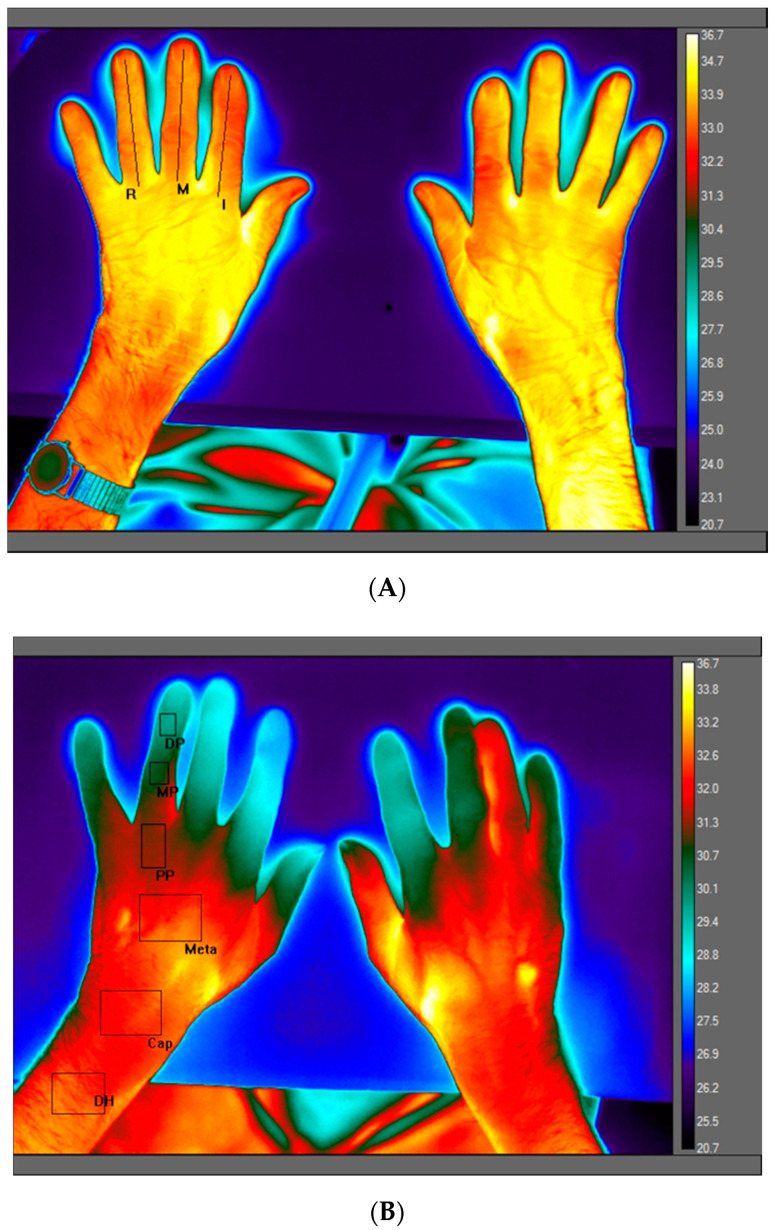
(**A**): Thermal map of left and right hand positioned upon an insulated tile. The region of interest (ROI, non-dominant hand) displayed as a vertical line constructed centrally for ring (R), middle (M) and index (I) fingers. Selection of the finger with greatest variability was used to construct six ‘box’ ROIs from which mean ROI values were obtained (B) and with data from the selected finger used in subsequent analyses. (**B**) shows anatomical ROI positions for temperature (°C) of distal phalange (T_DP_), middle phalange (T_Tmp_), proximal phalange (T_PP_), metacarpal (T_Meta_), capitate bones (T_Cap_) and distal humerus (T_DH_).

This provided visual orientation to the participants for the placement of fingers and hands in a consistent position and to obtain a clear field of view (FOV). After checking detector focus and distance, a maximum of three images were obtained. Colour thermal maps were obtained using a proprietary FLIR software (FLIR Systems AB, Täby, Sweden) and colour palette (see Figure 2).

In this paper, data were reported of the dorsum of the hand as this was the most comfortable position for older participants. The colour palette, with temperature key, shows darkest colours (indigo/blue) representing the lowest skin temperature and bright colour (white/yellow) highest temperatures (Figure 2).

#### 2.6.8. LWIR Region of Interest (ROI) and Image Processing

Emissivity was set to 0.98 for all images; temperature span 16 °C; range 20.7–36.7 °C. A ‘first-pass’ review of thermal maps for temperature variability across ring (R), middle (M) and index (I) finger was made. Thermograms showed that fingers typically appeared ‘non-uniform’ in temperature distribution. Thus, for each participant, a vertical ‘line’ ROI was constructed (Figure 2A) for each of R, M and I fingers. The finger with the greatest skin temperature value S.D (an indication of temperature variability) was used in subsequent analyses. For the selected finger, a series of six vertical ‘box’ ROIs (Figure 2B) were constructed (manually) traversing the centre of each finger of the non-dominant hand and using pre-defined dimensions; fingertip (distal phalange, T_DP_), wrist (capitate bones, T_Cap_) and forearm (distal humerus/ulnar T_DH_). Thee ROI dimensions were used throughout the post-processing of LWIR images; T_DP_, (280 pixels) T_Cap_, (2436 pixels) and T_DH_, (1900 pixels). On occasions, the anatomical ROI did not conform well to the finger (e.g., the hand was too slim for the size of the ROI or the hands were ‘gnarled’ by arthritis, preventing the ‘flat’ placement of the hands, so the ROI dimension was adjusted slightly to ‘fit’ ROI to finger anatomy. Mean temperature difference (°C) across the extremities were calculated as: T_DP_ − T_Cap_ (∆T_1_) and T_DP_ − T_DH_ (∆T_2_). The mean temperature difference between T_DP_ and tympanic temperature is given as ∆T_3_.

#### 2.6.9. Calibration

Calibration of the FLIR thermal camera was undertaken against a black-body source (P80P, Ametek-Land, Dronfield, UK) to determine temperature accuracy and thermal camera performance across a 20 °C environmental temperature range (20–40 °C).

#### 2.6.10. Warmth Sensation Rating

After adjusting to the study environment, each participant was asked to rate their thermal sensation using the 7-point thermal sensation scale with options ranging from −3 (cold), −2 (cool), −1 (slightly cool), 0 (neutral), +1 (slightly warm), +2 (warm), +3 (hot) [4]. The McIntyre scale [22] for thermal preference (thermal vote, TV) was used to obtain a response to the question “I would like to be”: options include (a) cooler (b) no change (c) warmer.

#### 2.6.11. Statistical Analyses

All computations have been carries out with SPSS version 24 (IBM, Armonk, NY, USA). The associated factors analysed in participants over 60 years are presented as numbers, percentage, mean (standard deviation, SD), with *p*-value. Chi-Square tests were used for categorical data. Anova tests were performed for comparison of mean values of independent variables amongst the two participant groups: ND and D. ANOVA was used to determine whether there were any statistically significant differences between the means of two independent groups (Table 1).

### 2.7. Ethics Approval, Screening and Recruitment

All subjects gave their informed consent for inclusion before they participated in the study. The study was conducted in accordance with the Declaration of Helsinki, and the protocol was approved by the East Midlands (Derby, UK) research ethics committee and Health Research Authority (HRA) (16/EM/0483).

## 3. Results

### 3.1. Characteristics of Older Adults Living in Residential Care

Seventy-three residents gave their consent to the study. Data was analysed from 69 people aged 60–101 (mean 84) years; 47 were female. Participants were recruited from 15 English residences. All were white British, Irish or European. Sixty residents had mild, moderate, or severe frailty. Nine residents only, were ‘well’ or ‘managing well’ on the frailty score. There was a significant difference concerning frailty with more residents in the dementia (D) group being severely frail (*p* = 0.028) (Table 1).

AMT ranged from 0–10 (median 8). Thirty-four residents were assigned to ‘dementia’ category, (D) and 35 residents, (ND) (Table 1). Residents were studied in groups of two to five at each imaging session (median three residents per session) in still air (<0.1 m·sec^−1^) T_a_ 21.4–26.6 °C (mean 23.6 °C) RH 32–78% (mean 51%). Under these indoor ambient conditions, a variety of clothing ensembles were worn (Clo range 0.26–1.54, mean 0.61 Clo) corresponding to light (*n* = 20) medium (*n* = 47) heavy (*n* = 2) clothing ensembles respectively.

### 3.2. Thermal Sensation (TS) Self-Rating

Of the 68 of 69 participants who provided a TS self-report, the majority rated the environment ‘comfortable’ (TS score 0, *n* = 43, 63%). For the remainder, responses raged from +2 (*n* = 3), +1 (*n* = 7), −1 (*n* = 13), −2 (*n* = 1) corresponding to ‘warm’, ‘slightly warm’, ‘slightly cool’, ‘cool’ respectively with a range of different TS ratings between residents across individual care homes (Table 2). None of the residents rated −3 (cold) or +3 (hot). Overall, 92% of residents rated TS −1 to +1. Most residents (*n* = 55, 80%) on providing a TV did not wish to change the temperature of the environment whereas eight (11%) would have preferred a warmer temperature and six (9%) a lower air temperature.

### 3.3. Thermal Sensation Ratings and Clothing Insulation

When sharing the same room (and thus same T_a_), differences in TS rating and TV were reported. A significant difference in TS rating was noted between residents (D vs. ND) (*p* = 0.02, Table 1). Residents (D) more frequently expressed feeling ‘slightly cool’ or ‘cool’ (*n* = 11) compared with ND residents (*n* = 3; Table 1). By contrast, residents without dementia more frequently expressed feelings of being ‘slightly warm’ or ‘warm’ (*n* = 9) compared to those with dementia (*n* = 2). Throughout the months during which the study was conducted, clothing worn by the majority of residents (Table 1) provided light to medium insulation; differences in clothing insulation tending towards medium to heavy Clo units for residents with dementia (borderline significance, *p* = 0.05).

### 3.4. Core (Tympanic) Temperature

Residents were afebrile, T_tymp_ 35.5–37.5 °C (mean 36.7 °C). A statistical but not clinically significant difference was observed between ND vs. D groups (*p* = 0.01, Table 1). Differences (T_DP_ − T_tymp_) were consistently negative for all older adults; ∆T_3_ range −12.5 °C to −2.3 °C.

### 3.5. Skin Extremity Temperature

**Figure 3:**
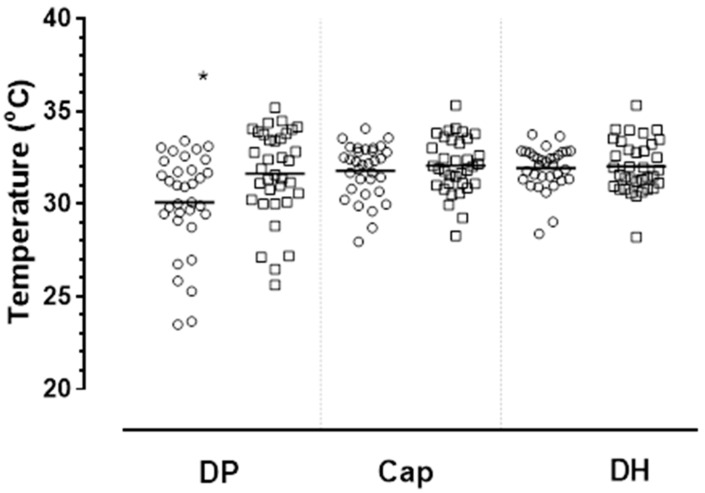
Mean skin temperature at each of three ROI regions represented by finger-tip (distal phalange, T_DP_), capitate bones (T_Cap_) and forearm at distal humerus/ulner (T_DH_) of residents with dementia/AMT < 8 (D) (open circles, O) and residents without a confirmed diagnosis of dementia/AMT ≥ 8 (ND) (open square □). * Significant difference between mean temperature for T_DP_ of residents D vs. ND group (*p* = 0.01). Horizonal bars represent mean values.

Right hand was dominant in 63 of 69 residents (91%). Thermal mapping and data analysis of ‘non-dominant’ finger/hand ROIs were therefore performed predominately for left hand. Individual extremity skin temperature values for T_DP_, T_Cap_, T_DH_ for all residents are lowest for T_DP_. Mean vales for T_DP_, T_Cap_, T_DH_ were 30.9 °C (2.6 °C), 31.9 °C (1.5 °C), 31.9 °C (1.3 °C) respectively. Mean T_DP_ was significantly lower (*p* = 0.01) for residents with dementia compared to ND (Table 1; Figure 3).

### 3.6. Comparisons Between Groups

**Figure 4:**
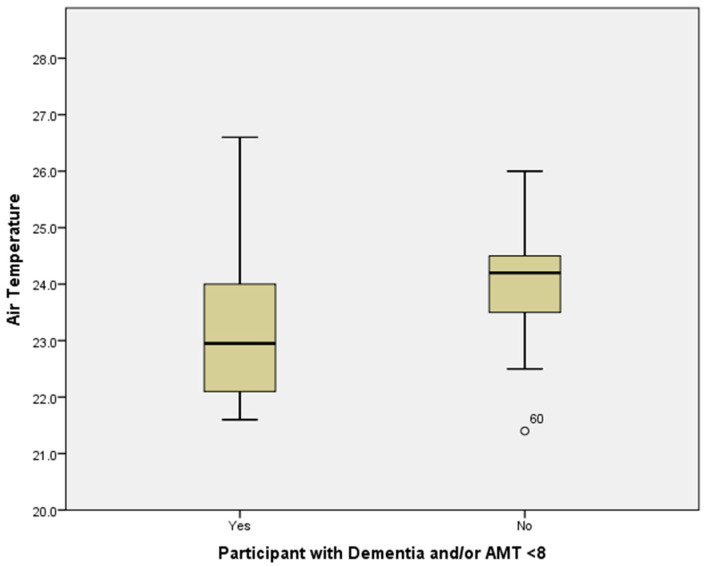
Distribution of air temperature (°C) within care homes by group: dementia or an AMT < 8 (yes/no) showing median, lower and upper quartiles, and lower and upper extremes of air temperature. Outlier: participant studied at lowest T_a_, 21.4 °C.

D vs. ND: Air, extremity skin and tympanic temperature: T_a_ of the communal rooms where residents were sitting was, on average, 1.0 °C lower (*p* = 0.001) in D compared to the ND group (Table 1; Figure 4).

**Figure 5:**
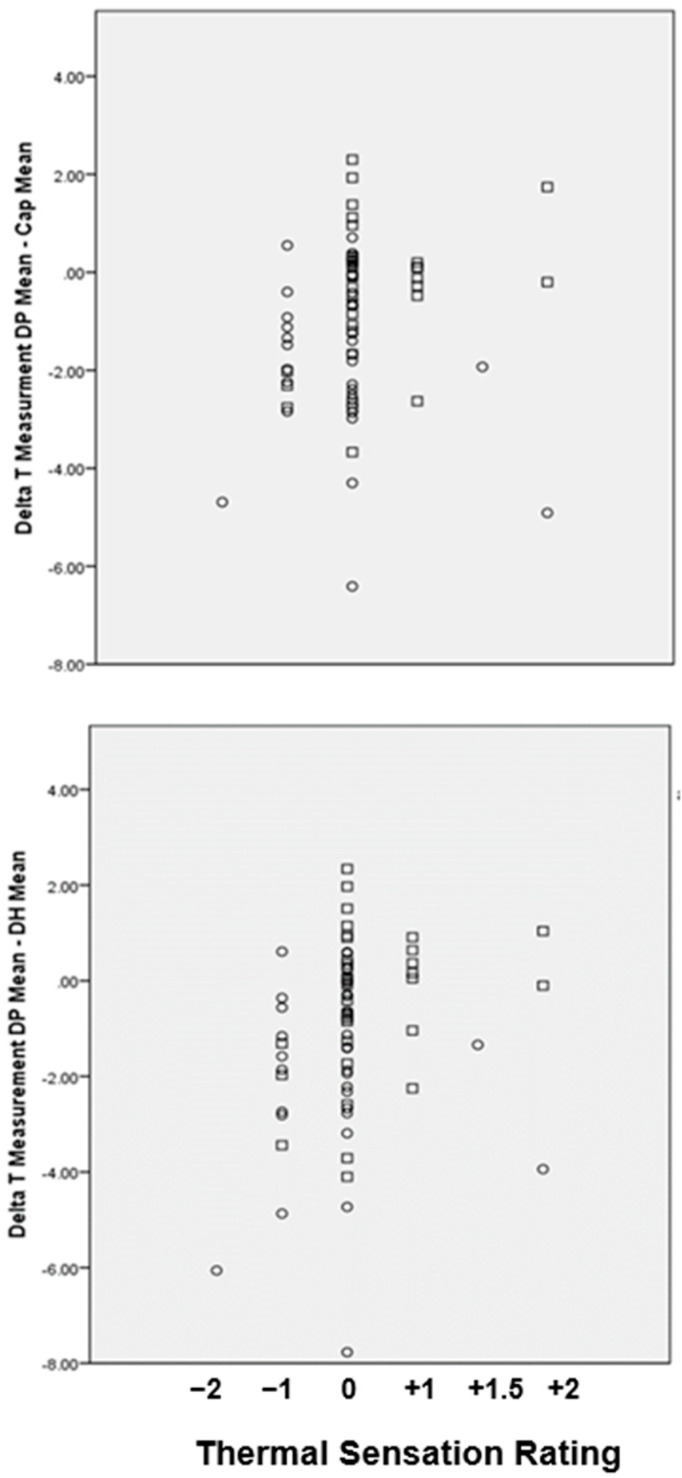
Upper panel: extremity temperature difference between fingertip to wrist ∆T_1_ (mean T_DP_- mean T_Cap_) and lower panel: fingertip to forearm, ∆T_2_ (mean T_DP_ − T_DH_) by group and TS ratings; dementia (O); no dementia (ND).

**Figure 6:**
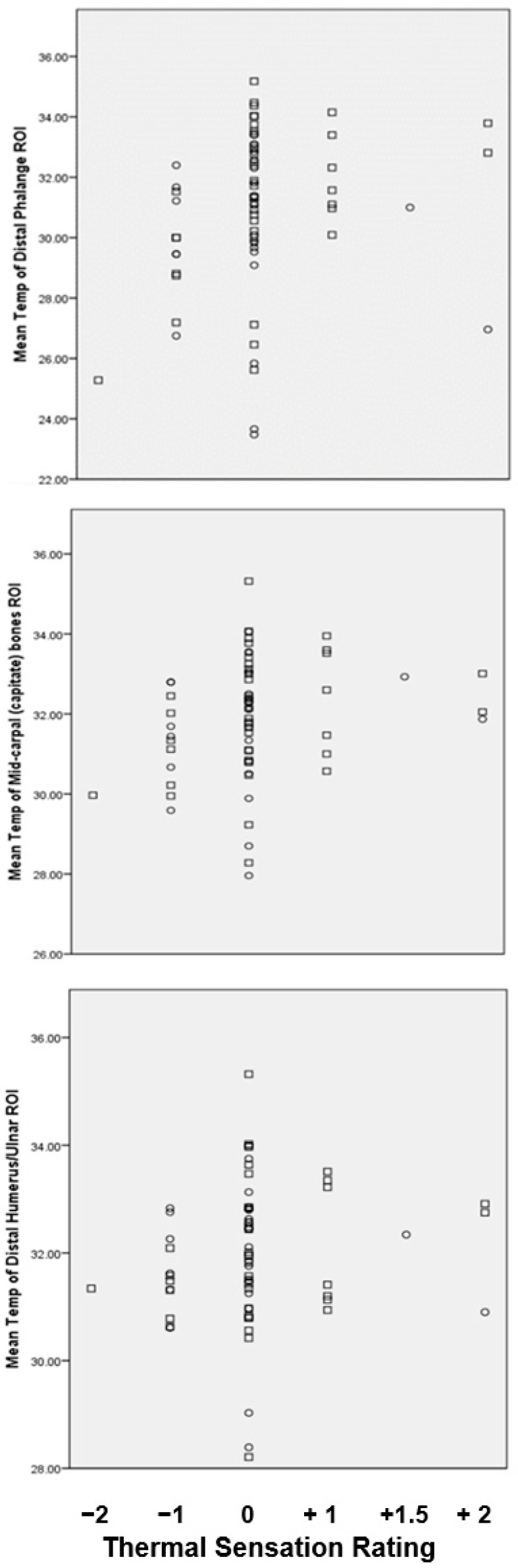
Mean extremity temperature values at three ROIs; distal phalange T_DP_ (upper panel); capitate bones, T_Cap_ (middle); distal humerus, T_DH_ (lower) by group and TS rating. dementia (O), no□ dementia (□) for each reported thermal sensation rating.

A wide range of ROI temperature differences were recorded at each ROI (Figure 5) and from these values the temperature gradient, delta T (∆T) calculated for ∆T_1_ (T_DP_ − T_Cap_) and ∆T_2_ (T_DP_ − T_DH_) with respect to TS rating. Figure 6 shows the mean temperature (°C) at each of the three ROIs with respect to each residents’ reported TS rating.

For ∆T_1_, mean difference, 0.43 °C (0.24) (ND) vs. −1.7 °C (0.29 °C) (D) *p* = 0.001. For ∆T_2_, mean difference, 0.39 °C (0.26 °C) (ND) vs. −1.8 °C (−0.34 °C) (D) *p* = 0.001 (Table 1) The range of temperature differences, ∆T_1_ for residents with dementia ranged from −6.4 °C to −1.5 °C and for residents without dementia, −3.7 °C to 2.3 °C. The range of temperature differences, ∆T_2_, −7.7 °C to 0.6 °C and −4.1 °C to 2.3 °C for D vs. ND respectively. A significant difference (mean 0.2 °C) for tympanic temperature (*p* = 0.01) was observed between groups ND and D.

### 3.7. Extremity Temperature Values and Thermal Sensation Rating

On further exploration of thermal sensation for T_DP_, residents who were dissatisfied with the environment provided ratings of −2 and −1 (slightly cool, cool, respectively), 11 were residents with dementia (D) and 4 did not have dementia (ND). Eleven residents (9 ND, 2 D) were also dissatisfied with the environment perceiving it as slightly warm (+1, +1.5) or warm (+2).

### 3.8. Thermal Mapping of Extremities: Correspondence Between the Thermal Map and Thermal Sensation Report

**Figure 7:**
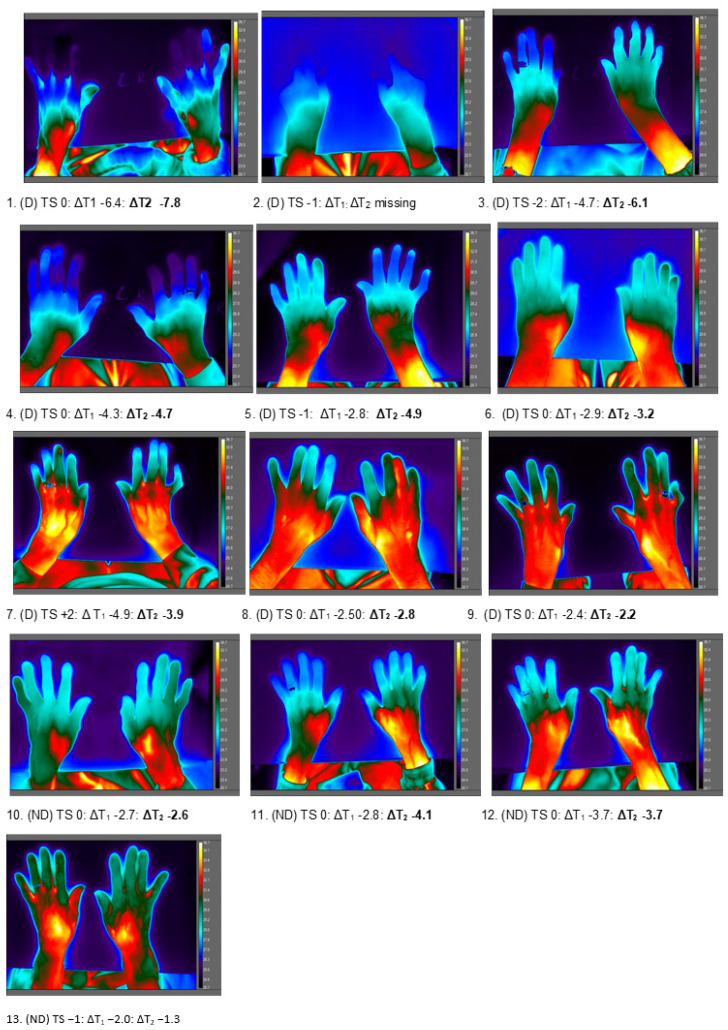
Thermal maps of hand and forearm of 13 residents with the visual appearance of ‘cold’ hands. Figure shows the study group; dementia (D) or no dementia (ND), thermal sensation (TS) rating and the individual temperature difference (°C) for ∆T_1_ and for ∆T_2_.

Qualitative review of hand thermograms, showed the visual appearance of skin temperature for ∆T_1_ and ∆T_2_ of all residents in the environment of their cluster groups:

*(A) ‘Cold hands’:* LWIR thermogram appearance was not consistent with thermal sensation report. Thirteen residents (9 in D category) showed the visible appearance of ‘cold hands’ mean T_DP_ < 30 °C (range 23.5–29.9 °C; median 26.5 °C) in air temperatures ranging from 21.4 °C to 25.6 °C (median 23.5 °C) (Figure 7). Thermal sensation ratings were variable ranging from −2 (cool *n* = 1), −1 (slightly cool, *n* = 3), 0 (comfortable/neutral, *n* = 8) to +2 (warm *n* = 1). For cold hands, the temperature difference for ∆T_1_ ranged from −6.4 °C to −2.0 °C (median −2.8 °C) and for ∆T_2_ −7.8 °C to −1.3 °C (median −3.8 °C). A clear demarcation in temperature across the hands was observed such that areas were ‘invisible’ against ambient temperature on the thermal map. ‘Thermal amputation’ was evident visually for the digits in 4 residents with (D) where ∆T_1_ was −6.4 °C, −4.7 °C, −4.3 °C (*n* = 1 data missing).

**Figure 8:**
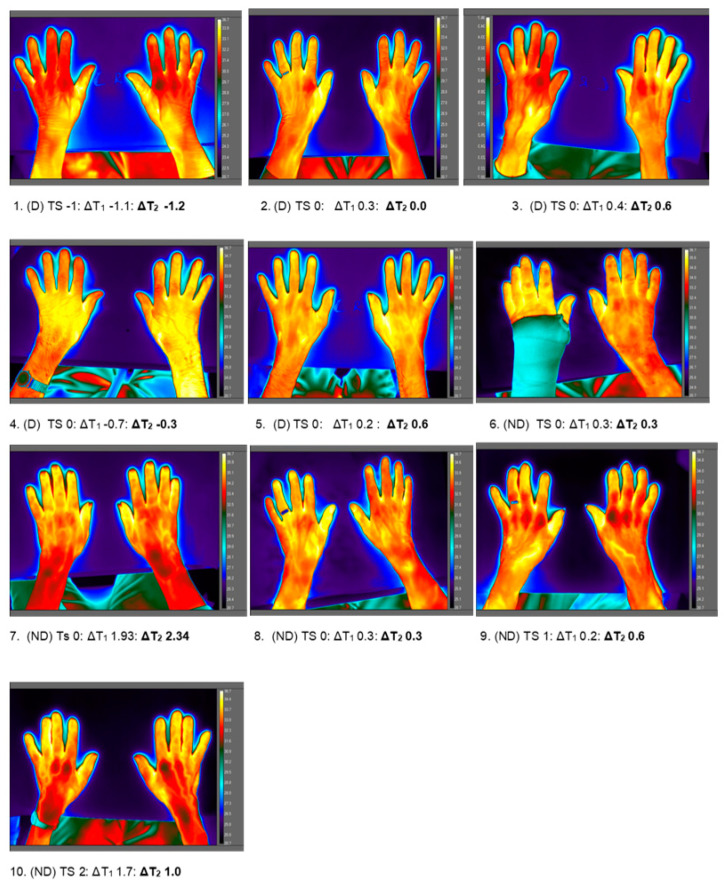
Thermal maps of hand and forearm of 10 residents with the visual appearance of ‘warm’ hands. Figure shows the study group; dementia (D) or no dementia (ND), thermal sensation (TS) rating and temperature difference (°C) for ∆T_1_ and for ∆T_2_.

*(B) ‘Warm hands’:* By contrast (Figure 8) the brightest colour appearance visually on thermograms corresponded with highest hand temperatures in 10 residents (5 D; 5 ND) (mean T_DP_ 31.7–35.2 °C; median 33.7 °C) across air temperature of 21.9 °C to 25.3 °C (median 22.9 °C) i.e., similar air temperature to those with cold hands. Individual thermal sensation ratings for this group were: −1 (slightly cool, *n* = 1); 0 (comfortable/neutral, *n* = 7), 1 (slightly warm, *n* = 1) and 2 (warm, *n* = 1). Temperature difference across the hands for ∆T_1_ ranged from −1.1 °C to +1.9 °C (median 0.3 °C). Similarly, for ∆T_2_, mean temperature differences between ROIs was small: range −1.2 °C to +2.34 °C (median 0.45 °C).

### 3.9. Medical History and Medications with Potential Influence on Perception of Temperature and Thermal Appearance

None of the residents had a past medical history of Raynaud’s disease. There was however, a significant difference in the number of current medications between the residents with and without dementia (*p* = 0.001). However, with respect to medication with known vasoconstrictor or vasodilator effects which might be expected to have an effect on the distribution of blood flow (and temperature) at the extremities, there was no significant difference between the groups, neither were there significant difference in the medications given with known effects on neurological function known to impact on perception of thermal comfort. No other significant difference in medication type was noted for the remaining classes of prescribed drugs.

## 4. Discussion

The majority of care home residents in this study were in the older-old age group; 27% aged 90 years or more and with approximately half of the group with cognitive deficits. Studies of thermal comfort are typically performed under controlled conditions of a climate chamber e.g., [13,23]. Whilst Soebarto et al. [24] did undertake studies (of young and old people) in a climate chamber in both young and older people, the authors comment that this is not practical for the very old and frail. Undertaking the current study under the ‘real-world’ conditions of the care home provides a true representation of an individual’s day to day indoor environment and associated thermoregulatory responses. In seeking to understand the responses of older people to their environment, we recognise that identifying those with cognitive deficits (vascular cognitive impairment, Alzheimer’s prodrome) often falls short of an exact medical diagnosis [25]. This makes disease classification for research [26] to two binary groups (‘dementia’ vs. ‘no dementia’) rather more ‘nuanced’ because age-related cognitive decline follows a continuum across the boundary between normal cognition to the severely demented [26]. It was therefore not possible to assign residents accurately to two groups based on diagnostic differentiation using neuropathology or amyloid biomarkers [27] so the AMT score was used as a pragmatic alternative in the absence of a confirmed diagnosis.

Conducted across 15 English residential care homes, the overarching finding was that older people exposed to the same environmental conditions sense thermal comfort quite differently from one another and this carriers an important message for their carers, alerting them to the potential that residents will experience both satisfaction and dissatisfaction under the same indoor conditions.

Thermal comfort is just one aspect of the environment that exerts an effect on older people [11,28] especially those with dementia [29]. It is clear from the work of Walker et al. [10] that during cold weather, carers and managers are concerned about keeping residents warm and comfortable but see this as challenging due to the diversity of co-morbid conditions, frailty, and different levels of activity of people who live together. The same concerns hold for keeping residents cool in hot weather [3]. However, in the absence of any established method or consensus on how best to provide thermal comfort for the majority, it is likely that those residents in thermal discomfort (whether too cold or too hot) will be overlooked, especially if they are unable to communicate effectively.

Many researchers [30,31,32,33] have explored skin temperature (the physiological interface with the environment) as a predictor of thermal comfort. For example Wu et al. [34] investigated upper skin extremity temperature (finger, wrist, hand, forearm) and the conditions required for indoor thermal comfort in an office environment using the same 7-point thermal sensation scale as for the current study. In mean air temperature of 26.8 °C, 60% of adults rated thermal sensation 0 (‘neutral’) and 90%, rated TS from −1 to +1 (slightly cool, neutral, slightly warm). Similar results were observed in the older aged residents in this study, 63% and 92% rating TS 0 (neutral) and −1 to +1 respectively, albeit in mean air temperature 2 °C lower than the office-based adults. As for upper extremity temperatures in the older residents, mean finger-tip (30.9 °C), wrist (31.9 °C) and forearm (31.9 °C) temperatures were 2 °C lower than reported by Wu et al. [34] where corresponding regions (fingertip, wrist, forearm temperatures) were 33.4 °C 33.7 °C and 33.7 °C respectively. Of interest therefore is that whilst older adults had lower mean skin temperatures and were in lower indoor temperatures, a comparable percentage of residents were satisfied with the environment and rated their TS as 0 (neutral/comfortable). The possibility that the older person’s thermal perception of the environment is ‘blunted’ is consistent with the biological consequences of ageing on thermogenesis and decline in thermosensitivity [35]. This blunting of thermal sensation can occur similarly as in other types of sensory perception loss with age [36]; hearing, vision, taste and smell being additional examples. We have observed features of thermal blunting in the older residents, particularly those with dementia where we observed residents with low extremity (digit) temperatures corresponding to ‘cold hands’ reporting thermal sensation as neutral (or comfortable) even with obvious visual ‘thermal amputation’ on the thermal map and even where environmental temperature was within the thermoneutral range [37,38].

In the thermoneutral zone, skin blood flow in the hands is tonically active and vasomotor tone of skin capillaries operates as the primary ‘controller’ of deep body temperature. Hands (and feet) represent ‘radiator’ organs [39] losing heat to the environment as well as retaining and conserving body heat. Skin temperature therefore varies with changes in vasomotor tone. Capillaries of non-glabrous skin, along with arterio-venous anastomoses (AVAs) of glabrous (hairless) skin of hands and feet are continuously adjusting (cycling) blood flow to extremity skin to balance heat loss with heat retention [38,40]. These physiological measures, independent of an individual’s TS perception may provide a more robust indicator of temperature derangement than achieved through thermal comfort scales, especially under conditions where there is a risk of ‘symptomless cooling’ [14,18]. If it is possible to measure and/or ‘see’ the consequences of marked vasoconstriction (or vasodilation) at the extremities this may provide a more reliable indicator of thermal risk; ‘cooling without noticing’. We have shown previously [8], as have others [41], that the feeling of being chilled tends to start in the hands or feet. Harazin et al. [42] report finger skin temperature (at a ‘cut-off’ temperature below 29 °C) in adults (with vibrotactile perception disorders consequent on peripheral neuropathy) as a characteristic of ‘cold hands’ even in air temperature above 21 °C.

In addition, Pathak et al. [23] have shown that skin temperature gradients are significantly related to resting metabolic rate such that air temperature of 25 °C may serve as an objective measure for the conditions to maintain homeothermy. Looking further at both extremity skin temperature, Wu et al. [34] report finger temperature above 30 °C (and finger-forearm temperature gradients close to 0 °C) to represent a significant threshold for an overall sensation of thermal comfort. Furthermore, at mean air temperature of 26.8 °C, Wu et al. [34] report temperature gradients between fingertip to wrist and fingertip to forearm ranging from −3.5 to 0.3 °C and −4.0 °C to −0.3 °C respectively; the negative temperature gradient serving as an indicator of a ‘cool’ TS response.

In the current study, older adults showed peripheral vasoconstriction as evidenced by the temperature gradients across the extremities; more intense in those with D than ND for both fingertip to wrist (∆T_1_) and fingertip to distal forearm (∆T_2_). At mean air temperature of 23 °C (Group D) and 24 °C (group ND) this air temperature is within the thermoneutral (comfort) zone (23–27 °C) for light to moderately clothed adults [23] yet older residents show evidence of extremes of thermoregulation as evidenced by both marked peripheral vasoconstriction and vasodilation in hand ROIs observable on the thermal maps.

Being able to take temperature measurements using conventional thermometry and across multiple areas of the skin surface in the setting of care homes presents a significant challenge in routine care. However, with infrared thermography, a quick visual assessment of the physiological response to the environment can be made by imaging of the extremities. Heat maps reflect the net effect of changes in vasomotor tone on skin temperature. As far as it is possible to tell, this is the first report of an independent imaging technology to map ‘what we see’ on LWIR thermography with ‘what people say’ about their thermal comfort. In other words, can we ‘see’ signs of thermal discomfort using thermal imaging and is there a potential benefit in doing so? Although other techniques; laser Doppler imaging [43], laser speckle imaging [44] or venous occlusion plethysmography [45] are available, they are rather less practical for the conditions of the care home whereas LWIR thermography offers an ‘at a glance’ imaging solution about thermal conditions of the ‘radiator’ organs, the most obvious exposed skin site being the hands. What we see on thermal imaging is the distribution of heat at the extremities which, at least for digit skin temperature (where metabolically active tissue is minimal) is entirely due to blood flow.

On qualitative review of thermal maps, the appearance of ‘cold’ and ‘warm’ hands emerged based on colour coding across a temperature span of 16 °C. For residents with ‘cold hands’, fingertip temperature (T_DP_) was, in all cases <30 °C with fingertips consistently colder than wrist and forearm and with a wide (negative) skin temperature gradients for ΔT_1_ and ΔT_2_ irrespective of TS rating. These results support the work of both Pathak and Wu [23,34] (albeit studying younger, healthy adults) that fingertip temperature below a cut-off, together with wide (negative) temperature gradient (fingertip-wrist and fingertip-forearm) occur across the thermoneutral range. As we have observed, on review of the hand thermograms, this powerful vasoconstrictor response, which can decrease skin blood essentially to zero [46], is not always accompanied by a sensation equivalent to thermal discomfort.

Whilst commonly used models for thermal comfort are based on Fanger’s work [47], such models were developed from studies in young, healthy adults in the workplace. That these models are inappropriate for older people is now recognized because changes in the structure of the nervous system as people age means that thermal sensitivity and perception decreases [14]. In the older group of residents, we have seen how varied the thermal comfort responses of older people are, even under the same environmental temperature. Shahzad et al. [48] have shown that neutral thermal sensation does not guarantee thermal comfort; 36% of participants in their study did not want to feel ‘neutral’ as their comfort condition, preferring a non-neutral thermal sensation. This finding supports the work of de Dear [49] in differentiating thermal ‘pleasure’ from thermal ‘neutrality’; some people finding a cool environment more ‘pleasing’ than a neutral position which, apart from personal preference may also be influenced by cultural and social factors. For example, Florez-Duquet et al. [50] showed that older subjects generally did not report, or complain, of cold even during an entire cold exposure test whereas young adults did. Taylor et al. [15] showed that older people require a more intense stimulus, starting at the extremities before they ‘feel’ cold. Consequently, older people are being exposed to intense thermoregulatory challenges that will go unnoticed under the ‘normal’ indoor temperatures of the care home.

As the focus for long-term care has shifted from processes of care (safety, medical concerns) towards improving outcomes for residents [1] opportunities arise to meet this new challenge; to improve quality of life through considerations of the care home environment [51].

Whilst the majority of residents (both groups), expressed satisfaction with the environment by rating 0 on the thermal sensation scale, many did not; rating the environment too cool or even too warm. The first impression therefore, would be that residents were not in their comfort zone even in warm conditions but is this truly a measure of true satisfaction with the environment? Further evidence of the validity of the thermal sensation report can be explored by investigating concomitant changes in physiological factors, notably the degree of peripheral skin vasomotor tone.

Finally, of importance, in the context of determining the health and thermal comfort of older people in residential care, is not only in the ability to spot the vulnerable person at risk of chilling (or overheating) but in finding a practical solution to the variability in thermal sensation responses to the indoor environment in this older population, many of whom are immobile. Personal thermal comfort approaches could include ‘smart’ garments and local climate ‘bubbles’. Our next step will be in tackling the best approaches to determine a range of approaches that are practical and feasible within the care home. What is clear, due to the COVID-19 pandemic, is that assessing residents by touch will now be excluded for any thermal comfort assessment for the near future.

## 5. Conclusions

What we have observed by undertaking this feasibility study, perhaps more useful to those involved in the care of older people than relying on a persons reported thermal sensation rating, is in being able to ‘see’ the physiological responses to the environment in which they live. It is recognised that in older age, cultural factors as well as decline in neurosensory function can have an impact on sensory perception such that these senses may be blunted. This further confounds the value of thermal comfort rating scales in older people. The quick, relatively inexpensive, technique of thermal imaging, allows an immediate assessment of ‘live’ efferent thermoregulatory activity without the need for absolute measurements per se, so providing a new aspect of multi-dimensional thermal assessment. Thus, from the physiological ‘first responders’ of thermoregulation: skin extremity temperature (and concomitant extremity skin perfusion), the technique of infrared thermography could, in the future, provide technology-driven approaches to thermal assessment. Thermographic mapping of extremities as a prodromal thermal signature of incipient chilling (or overheating) could offer a better biomarker of thermal satisfaction and temperature safety within the environment than an older person’s own temperature sensibility. The future for this challenging field of health care will be in designing solutions to promote personalised thermal comfort involving interdisciplinary collaborations across medical, engineering, design and the built environment.

## Figures and Tables

**Table 1:** Characteristic of care home residents, clothing ensemble, ambient conditions and core and skin temperature values (°C) with temperature difference (ΔT). Table shows descriptive analysis that focused on associations between key variables.

Categorical Factor	Dementia Diagnosis/AMT < 8 Group D *n* = (%)		AMT 8–10 Group ND *n* = (%)		*p* Value
Male	10 (45.5)		12 (54.5)		0.43
Female	24 (51.5)		23 (48.9)		
**Age: years**					
60–70	2 (33.3)		4 (66.7)		0.25
71–80	9 (69.2)		4 (30.8)		
81–90	16 (51.6)		15 (48.4)		
Over 90	7 (36.8)		12 (63.2)		
**Ethnicity**					
White British	33 (50)		33 (50)		0.22
White European	0		2 (100)		
White	1 (100)		0		
**Frailty Score**					
Well	0		1(100)		0.028
Managing Well	6 (75)		2 (25)		
Vulnerable	1 (10)		9 (90)		
Mildly Frail	1 (50)		1 (50)		
Moderately Frail	9 (40.9)		13 (59.1)		
Severely Frail	17 (65.4)		9 (34.6)		
**Dominant Hand**					
Right	31 (49.2)		32 (50.8)		0.4
Left	3 (60)		2 (40)		
Ambidextrous	0		1 (100)		
**Finger with greatest SD**					
Left ring	11 (68.8)		5 (31.2)		0.34
Left middle	5 (38.5)		8 (61.5)		
Left index	14 (43.8)		18 (56.2)		
Right ring	1 (100		0		
Right middle	0		1 (100)		
Right index	2 (40)		3 (60)		
**Clothing ensemble (Clo unit)**					
Light	6 (30)		14 (70)		
Medium	26 (55.3)		21 (44.7)		0.05
Heavy	2 (100)		0		
**Thermal sensation rating**					
−2	1 (100)		0		0.02
−1	10 (76.9)		3 (23.1)		
0	21 (48.8)		22 (51.2)		
1	0		7 (100)		
1.5	1 (100)		0		
2	1 (33.3)		2 (66.7)		
**Thermal Vote**					
Cooler	1 (16)		5 (84)		0.09
No change	27 (49.1)		28 (50.9)		
Warmer	6 (75)		2 (25)		
**Temperature**	*n* = (%)	Mean (SD)	*n* = (%)	Mean (SD)	
Air temperature (°C)	34 (49.3)	23.1 (0.2)	35 (50.7)	24.1 (0.17)	0.001
Relative Humidity (%RH)	34 (49.3)	49 (1.1)	35 (50.7)	52.1 (1.6)	0.2
ROI: mean DP (°C)	33 (48.5)	30.0 (0.46)	35 (51.5)	31.6 (0.4)	0.01
ROI: mean Cap (°C)	33 (48.5)	31.7 (0.25)	35 (51.5)	32.0 (0.25)	0.4
ROI: mean DH (°C)	33 (48.5)	31.9 (0.19)	35 (51.5)	32.0 (0.24)	0.1
ΔT_1_ (mean DP- mean CAP) °C	33 (48.5)	−1.7 (0.29)	35 (51.5)	−0.43 (0.24)	0.001
ΔT_2_ (mean DP-mean DH) °C	33 (48.5)	−1.8 (0.34)	35 (51.5)	−0.39 (0.26)	0.001
Tympanic temperature (°C)	34 (50)	36.6 (0.07)	34 (50)	36.8 (0.6)	0.01

ROI—region of interest; DP—distal phalange; CAP—capitate bones; DH—distal humerus.

**Table 2:** Individual thermal sensation ratings reported by residents sharing the same environmental conditions at each imaging session in groups of two to five. Study undertaken over a period of 12 calendar months at 15 residential care sites. Residents with dementia who participated in the imaging sessions are identified by highlighted and emboldened text. Four residential care sites (6,9,11,12) were visited twice.

Site ID	Month/Day of Study	Mean T_a_ (°C)	Mean RH %	Thermal Sensation Rating				
				Resident 1	Resident 2	Resident 3	Resident 4	Resident 5
1	7 June	24.3	60	**0**	missing	0		
2	13 July	24.0	47	0	0	0	−1	
3	12 July	24.3	56	0	1	1	1	
4	28 July	22.5	54	0	0			
5	6 September	24.6	60	0	0	0		
6	28 September	25.8	52	0	1	1	0	
6	6 October	23.6	38	0	−1	0	**0**	
7	12 October	23.1	57	**2**	**0**			
8	20 October	24.2	60	0	1	0	0	
9	7 November	22.0	53	**−1**	**−1**			
9	8 December	24.0	32	1	0			
10	11 April	22.0	55	**0**	**1.5**			
11	17 April	24.5	48	**−1**	**0**	**0**		
11	24 April	21.8	44	**0**	**0**	**−1**		
12	11 May	25.3	37	**−1**	**0**			
13	16 May	22.6	45	**0**	**0**	**0**	0	
14	18 May	24.2	46	**0**	**0**	**0**		
12	21 May	22.7	50	−1	0	**0**	**−1**	**−1**
11	12 June	22.0	53	**−1**	**0**	**0**		
15	14 June	23.6	54	**0**	**−2**	**−1**	0	2
11	19 June	22.1	54	**0**	**−1**	**0**		
6	19 June	24.0	58	0	2			

RH—relative humidity, emboldened text within highlighted cells indicates the residents with a confirmed diagnosis of dementia or Abbreviated Mental Test (AMT) < 8.

## Abbreviations

AMT	Abbreviated Mental Test
ASHRAE	American Society of Heating, Refrigerating and Air-Conditioning Engineers
CIT	Cold induced thermogenesis
Clo	clothing insulation unit
CRN	clinical research network
Icl	overall insulation of assembly in Clo units (Clo)
FOV	field of view
LWIR	long wave infrared thermography
PMH	past medical history
RH	relative humidity
ROI	region of interest
SD	standard deviation
T_a_	air temperature
T_r_	rectal temperature
T_DP_	distal phalange skin temperature
T_CAP_	capitate bones skin temperature
T_DH_	distal humerus/ulnar skin temperature
T_tymp_	tympanic membrane temperature
TV	thermal vote
Δ	delta
ΔT	temperature difference
UK	United Kingdom of Great Britain and Northern Ireland
TC	thermal comfort
TS	thermal sensation
